# Case Report: A Novel Heterozygous *ZP3* Deletion Associated With Empty Follicle Syndrome and Abnormal Follicular Development

**DOI:** 10.3389/fgene.2021.690070

**Published:** 2021-05-19

**Authors:** Yongzhe Chen, Zesong Wang, Yueren Wu, Wenbin He, Juan Du, Sufen Cai, Fei Gong, Guangxiu Lu, Ge Lin, Can Dai

**Affiliations:** ^1^School of Basic Medical Science, Central South University, Changsha, China; ^2^National Health Commission Key Laboratory of Human Stem Cell and Reproductive Engineering, Central South University, Changsha, China; ^3^Department of Basic Medicine, School of Medicine, Hunan Normal University, Changsha, China; ^4^Reproductive and Genetic Hospital of China International Trust Investment Corporation Xiangya, Changsha, China; ^5^Clinical Research Center for Reproduction and Genetics in Hunan Province, Changsha, China; ^6^National Engineering and Research Center of Human Stem Cell, Changsha, China

**Keywords:** *ZP3* mutation, empty follicle syndrome, zona pellucida, female infertility, follicular development

## Abstract

**Background:** Empty follicle syndrome (EFS) is defined as the complete failure to retrieve oocytes after ovarian stimulation. Although several mutations in *ZP1, ZP2, ZP3*, and *LHCGR* have been identified as genetic causes of EFS, its pathogenesis is still not well-understood.

**Methods:** Whole-exome sequencing (WES) was employed to identify the candidate pathogenic mutations, which were then verified by Sanger sequencing. A study in CHO-K1 cells was performed to analyze the effect of the mutation on protein expression. Additionally, immunohistochemistry (IHC) staining was used to examine follicular development and zona pellucida (ZP) assembly in the ovary of an EFS patient.

**Results:** A novel heterozygous deletion in *ZP3* (c.565_579del[p.Thr189_Gly193del]) was identified in the EFS patient. It was inherited dominantly and resulted in significant degradation of the ZP3 protein. Oocytes with degenerated cytoplasm and abnormal ZP assembly were observed in follicles up to the secondary stage, and many empty follicle-like structures were present.

**Conclusion:** We identified a novel *ZP3* mutation that expands the mutational spectrum associated with human EFS. We also showed the abnormal follicular development and ZP assembly of the EFS patient with the heterozygous ZP3 mutation, which provides new insights into the pathogenesis of EFS.

## Introduction

Female infertility has become an increasingly prominent problem. Approximately 50 million women around the world suffer from infertility (Mascarenhas et al., 2012). Regular ovulation and healthy oocytes are the basis of human reproduction. With the development of *in vitro* fertilization (IVF) as a treatment for infertility, we have been able to retrieve oocytes and discover their defects *in vitro*.

Empty follicle syndrome (EFS), first reported in 1986 (Coulam et al., 1986), is commonly defined as the complete failure to retrieve oocytes after ovarian stimulation for IVF, despite normal follicular development and appropriate serum estradiol level (Revelli et al., 2017). Follicle atresia and oocyte degeneration due to ovarian aging may be possible causes (Awonuga et al., 1998). In recent years, mutations in four genes, including *LHCGR, ZP1, ZP2*, and *ZP3*, have been linked to EFS (Yuan et al., 2017; Chen et al., 2018; Dai et al., 2019a; Lu et al., 2019; Yang et al., 2020; Wang et al., 2021). Nevertheless, the pathogenesis of EFS, especially in patients with gene mutations, remains largely unknown.

During IVF treatment, cumulus–oocyte complexes (COCs), which consist of granulosa cells surrounding the centrally located oocyte, are recovered from the individual's follicular fluid (Veeck, 1988). Each oocyte is encapsulated in a zona pellucida (ZP), an extracellular matrix that plays important roles in the communication between oocyte and granulosa cells (GCs) (Fagbohun and Downs, 1990), fertilization (Pang et al., 2011; Avella et al., 2014), and protection of the early embryo (Hasegawa and Koyama, 2007).

The human ZP comprises four glycoproteins encoded by *ZP1–ZP4* genes (Lefievre et al., 2004). In recent years, mutations in *ZP* genes have been reported to be responsible for oocyte anomalies and female infertility (Yang et al., 2020). An autosomal recessive truncating variant in *ZP1* was identified in a consanguineous Chinese family, and this was suggested to explain infertility in females with abnormal oocytes lacking a ZP (Huang et al., 2014). Chen et al. was the first to identify a heterozygous recurrent missense mutation in *ZP3* as the cause of EFS. This mutation exerts its effect via a dominant negative effect on the interaction of ZP proteins, thereby possibly affecting ZP assembly, preventing communication between the cumulus cells and the oocyte, and eventually leading to oocyte degeneration (Chen et al., 2017). In the past 3 years, 25 *ZP1* mutations, two *ZP2* mutations, and two *ZP3* mutations were identified in patients with EFS (Dai et al., 2019; Yang et al., 2020; Wang et al., 2021; Zhang et al., 2021).

In the present study, we identified a novel heterozygous *ZP3 deletion* associated with EFS and female infertility, which was inherited dominantly in a Chinese family. We also assessed the EFS patient's follicular development and ZP assembly by using ovarian sections and immunohistochemical staining (IHC).

## Case Presentation

### Clinical Characterization

**Figure 1:**
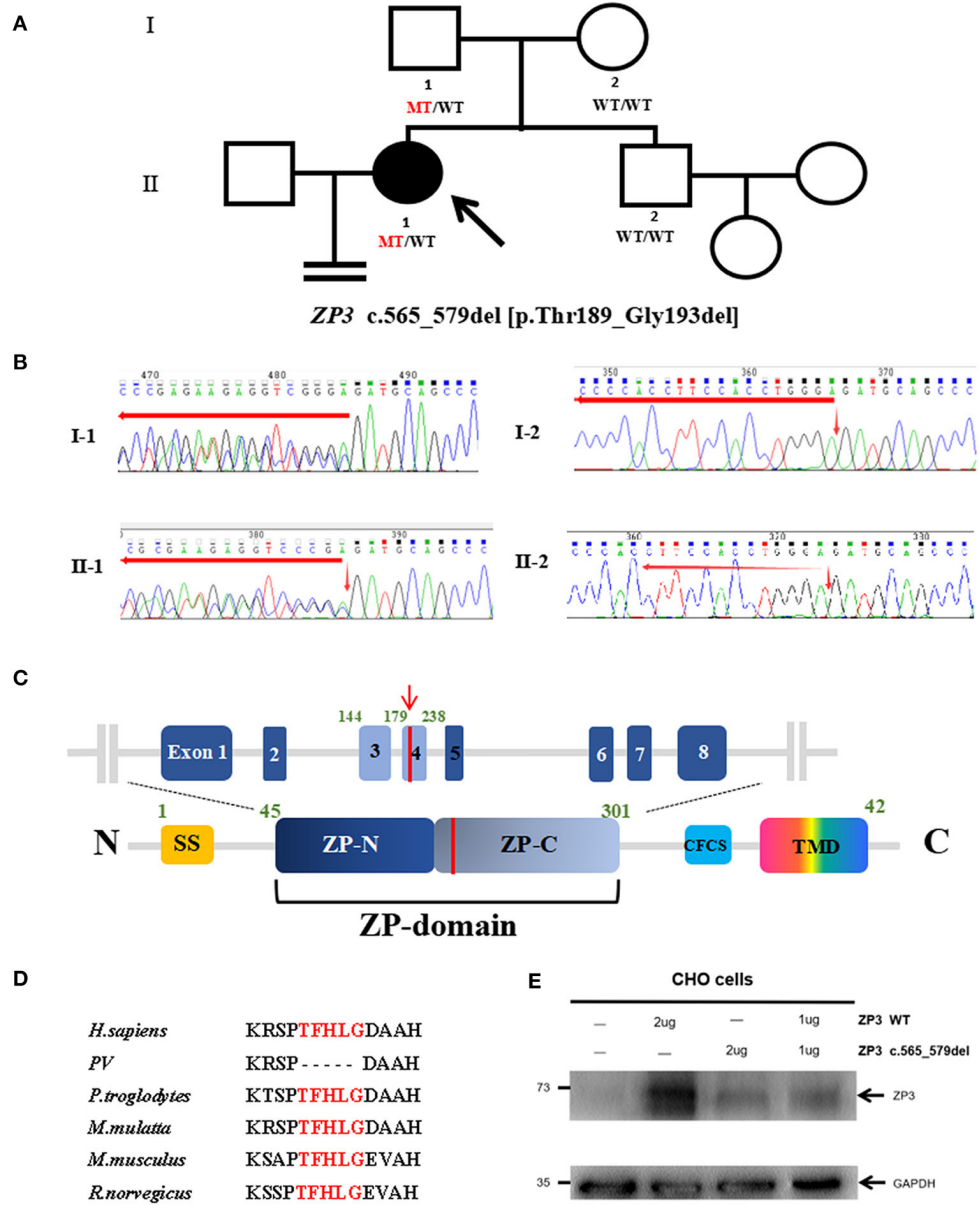
Genotypic features of the EFS patient. **(A)** Pedigrees of the family with EFS and female infertility. The filled circle indicates the EFS patient. The arrow indicates the proband. The ZP3 genotype for each subject detected is shown, with WT indicating a normal allele and MT indicating the mutant allele. **(B)** Sanger genotype of ZP3 for each individual. **(C)** Location of the mutation (red line) is indicated in the genomic structure (top) and domain organization (bottom) of ZP3. SS, signals sequence; CFCS, consensus furin cleavage site; TMD, transmembrane domain. **(D)** Thr189_Gly193 is highlighted in red and conserved in the selected species. **(E)** Expression of wild-type (WT) and mutant ZP3 proteins in Chinese hamster ovary (CHO) cells examined by immunoblot analysis using anti-ZP3 antibodies.

A patient affected by EFS (Figures 1A,B) was recruited from the Reproductive and Genetic Hospital of CITIC–Xiangya. She was 37 years old, with a diagnosis of primary infertility for 7 years. She underwent three failed artificial insemination (AI) treatments and four cycles of ovarian stimulation and oocyte retrieval in another hospital, in which no oocytes but three empty COCs containing small ooplasm-like fragments without ZP structure were obtained (Table 1).

**Table 1:** Clinical features of the EFS patient with a heterozygous *ZP3* deletion.

**Basal hormones**					
hFSH	(3.03–8.08 mIU/mL)			4.68	
hLH	(1.80–11.78 mIU/mL)			3.39	
E_2_	(21–251 pg/mL)			56	
Prog	(<0.1–0.3 ng/mL)			0.17	
Testo	(0.38–1.97 ng/mL)			0.56	
PRL	(5.18–26.53 ng/mL)			6.26	
**Clinical features with COH cycles**					
Cycle	1	2	3	4	5
	(other center)	(other center)	(other center)	(other center)	(our center)
Protocol	Ultra-long	Short	Ultra-long	GnRH antagonist	GnRH antagonist
Initial dose of Gn (IU)	225	225	225	NA	300
Duration of Gn days	4	8	9	NA	8
No. of COC retrieved	0	0	0	3	0
				Empty COCs with ooplasm-like fragments	

*hFSH, human follicle-stimulating hormone; hLH, human luteinizing hormone; E_2_, estradiol; Prog, progestin; Testo, testosterone; PRL, prolactin; Gn, Gonadotropins; COC, cumulus–oocyte complex; NA, not available*.

In our hospital, she received the fifth ovarian stimulation by the GnRH antagonist protocol. The rFSH/HMG was administered at a dose of 300 IU on day 2 of the menstrual cycle, and 300 IU per day for 8 days. Considering her history of recurrent failures and degenerated oocytes without ZP in the follicular aspirate, oocyte retrieval was performed by laparoscopy, upon identification of two follicles measuring 15.5 mm and 18.0 mm in diameters. Her serum estradiol level was 501 pg/mL. Unfortunately, no oocyte was obtained (Table 1).

### Identification of *ZP3* Mutation

The recurrent EFS in the patient led us to consider a genetic cause. To identify the causative mutation, we initially performed whole-exome sequencing (WES) and variant analysis in the proband and her mother. The following mutations were considered as candidate mutations: (1) mutations detected in the proband but absent in her mother; (2) mutations with a minor allele frequency below 1% in three public databases (1000 Genomes, the ExAC, and gnomAD Browsers); (3) exonic non-synonymous or splice site variants, or coding INDELs; and (4) mutations with high gene expression (reads per kilobase million ≥ 30) in human oocytes in both the previously described (Yan et al., 2013) and in-house RNA sequencing data. These criteria led us to identify a heterozygous deletion c.565_579del [p.Thr189_Gly193del] in *ZP3*. This mutation is highly conserved among species (Figure 1D) and is located within the ZP domain (ZPD) of the ZP3 protein, a structural module essential for the polymerization and assembly of ZP proteins (Figure 1C) (Jovine et al., 2002). To confirm the c.565-579del and exclude the possibility of a heterozygous deletion of a large fragment in *ZP3*, the full-length ZP3 cDNA was amplified for Sanger sequencing using total RNA from the peripheral blood and primers: sense 5′- CCTCCTGCTCTGGGGTAGTA-3′ and antisense 5′- CTTTTATTCGGAAGCAGACACAG-3′. As a result, the proband and her father harbored the same heterozygous deletion c.565-579del, while both her mother and brother had the wild-type, suggesting that the deletion had been paternally transmitted (Figures 1A,B) and had not affected male fertility. The karyotype of the proband was 46, XX.

### Effects of *ZP3* Mutation on Protein Expression

To test the effect of this *ZP3* mutation, expression plasmids carrying wild-type or mutant *ZP3* cDNA were transfected into Chinese hamster ovary (CHO-K1) cells and immunoblot analyses were performed by using primary antibodies ZP3 (1:500; Santa Cruz Biotechnology, sc-25802) and GAPDH (1:3000; Beyotime, AF0006), respectively. Antibody binding was detected with HRP-conjugated goat anti-rabbit (1:2000; CWBIO, CW0103S, for primary antibody of ZP3) and goat anti-mouse (1:2000; CWBIO, CW0102S, for primary antibody of GAPDH) secondary antibodies. The expression level of the mutant ZP3 protein was significantly lower than that of the normal protein (Figure 1E), suggesting that the mutant protein was unstable and degraded. Moreover, in the experiment of co-transfection of wild-type and mutant plasmids, simulating heterozygous mutation in the patient, the total level of ZP3 protein was as low as in the cells transfected with the mutant plasmid (Figure 1E), indicating a dominant negative effect of the mutant ZP3.

### Follicular Development in the EFS Patient With *ZP3* Mutation

**Figure 2:**
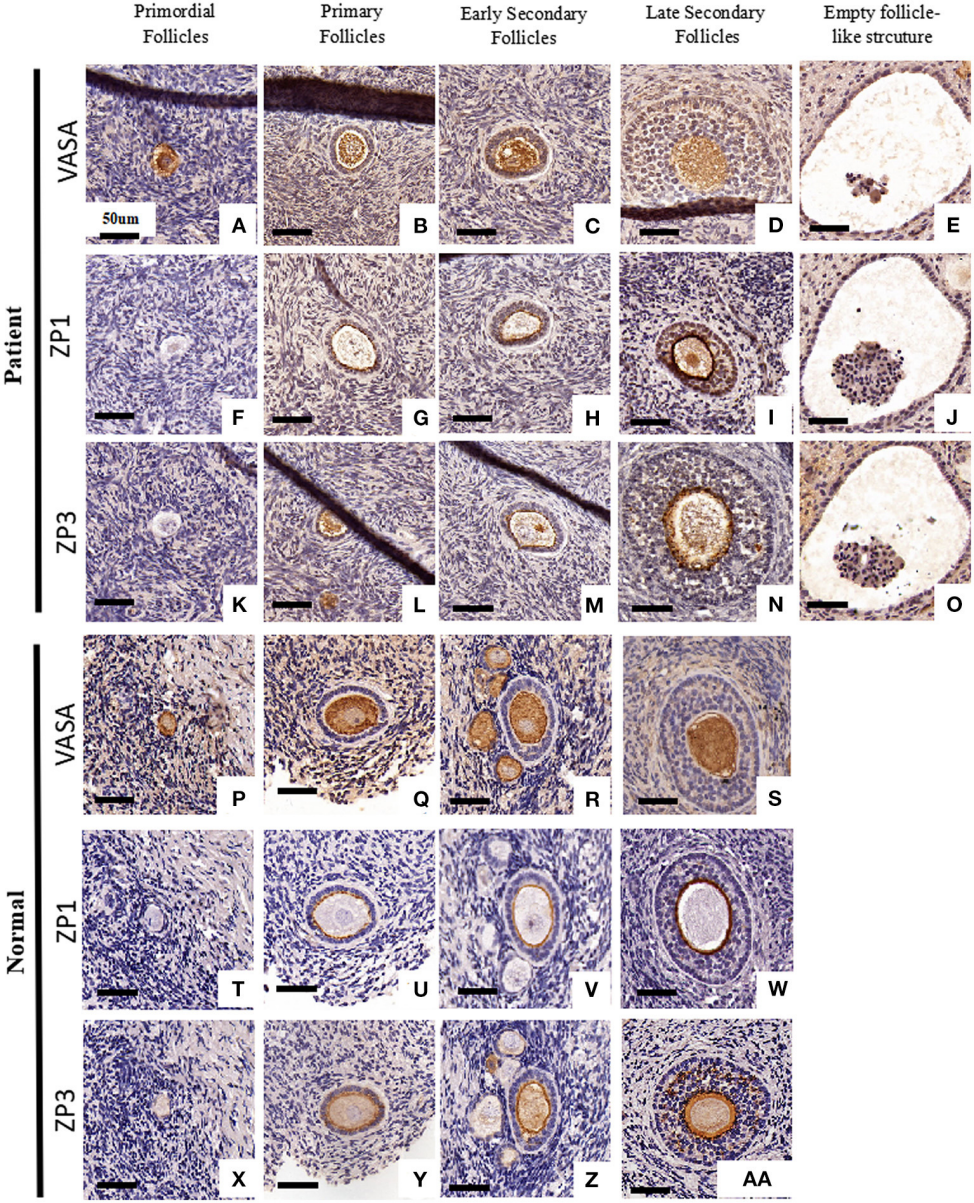
Ovarian histology and immunohistochemistry staining of the EFS patient with a heterozygous *ZP3* deletion. Ovaries isolated from the EFS patient carrying a *ZP3* mutation **(A–O)** and a normal control **(P–Z, AA)** were sectioned and stained with antibodies against VASA **(A–E, P–S)**, ZP1 **(F–J, T–W)**, or ZP3 **(K–O, X–Z, AA)**. The scale bars represent 50 μm.

**Figure 3:**
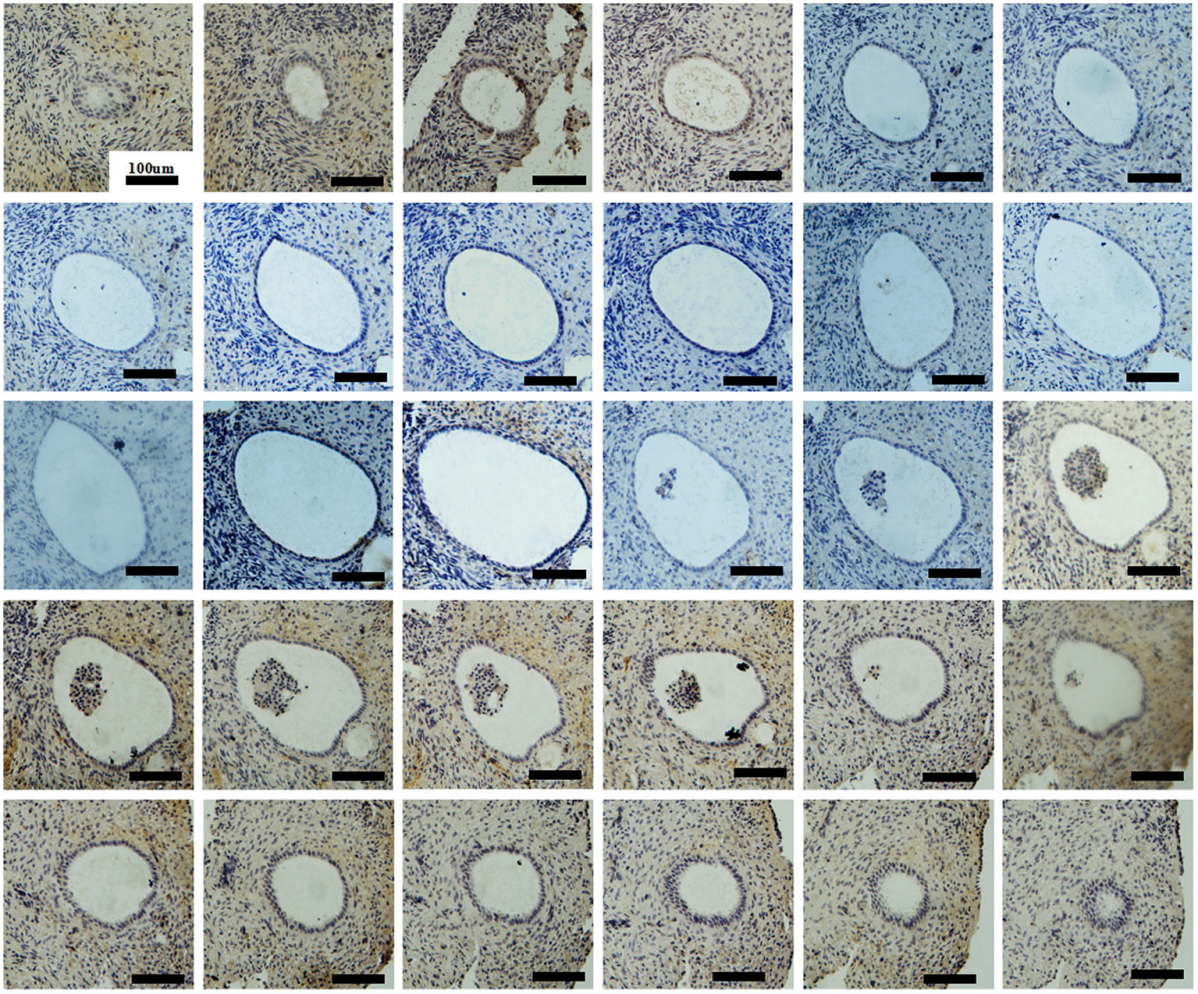
Ovarian histology and immunohistochemistry staining of serial sections showing an intact empty follicle-like structure of the EFS patient with a heterozygous *ZP3* deletion. There are a total of 30 sections with 5 μm per section and 223 μm in its widest part.

To examine follicular development and ZP assembly in the EFS patient carrying *ZP3* mutation, IHC staining on the ovarian sections was performed. Control ovarian tissue was obtained from the ovarian wedge resection of an individual with polycystic ovary syndrome (PCOS). Antigen retrieval was performed using 0.01 M sodium citrate buffer for 10 min, then endogenous peroxidase activity was blocked with 0.3% H_2_O_2_ in PBS for 15 min, and non-specific binding sites were blocked with 5% BSA in PBS at room temperature for 1 h. Sections from the same follicle were then incubated overnight at 4°C with primary antibodies to VASA (1:100, Abcam, ab13840), ZP1 (1:100; Santa Cruz Biotechnology, sc-365435), and ZP3 (1:100; Santa Cruz Biotechnology, sc-398359), respectively. Antibody binding was detected with HRP-conjugated goat anti-rabbit (1:1000; Abcam, ab97051, for primary antibody of VASA) and goat anti-mouse (1:1000; Abcam, ab97245, for primary antibodies of ZP1 and ZP3) secondary antibodies. The sections were then developed using DAB kit and counterstained with hematoxylin. Using VASA staining, intact follicles containing an oocyte at the primordial to secondary stages were observed in the patient's ovary (Figures 2A–D). It is worth noting that, compared with homogeneous cytoplasm in normal oocytes (Figures 2P–AA), the patient's oocytes showed meshy cytoplasm (Figures 2D,I,N), which suggested that the oocytes might be undergoing degeneration. Using ZP staining, a continuous ZP structure increasing in width along with growing oocyte was observed in the normal control (Figures 2T–AA). In contrast, no clear and intact ZP structure was detected in the patient's secondary follicles (Figure 2N). Moreover, no follicles beyond secondary stage were found, but many empty follicle-like structures were detected (Figures 2E,J,O, 3). These results hint the abnormal ZP assembly and follicular development beyond the secondary stage in the EFS patient with *ZP3* mutation.

### Discussion

Since 2014, dozens of mutations in human *ZP* genes have been identified as the cause of oocyte anomalies and female infertility. The mutations in *ZP* gene are the predominant causes of EFS (Yang et al., 2020). Most *ZP* mutations in EFS patients have been identified in *ZP1*, while only two were found in *ZP2* and *ZP3*. Here, we performed genetic analysis on a Chinese woman who was diagnosed with primary infertility and EFS, considering that oocytes could not be obtained in five cycles of oocyte retrieval following ovarian stimulation. By using WES and Sanger sequencing, we identified a novel heterozygous deletion of *ZP3* inherited in an autosomal dominant pattern. This is in agreement with the earlier studies that identified two heterozygous missense mutations in *ZP3* inherited dominantly in four independent EFS families. Our functional studies showed that this heterozygous *ZP3* deletion led to the degradation of both the mutant and wild-type ZP3 proteins.

The human ZP is composed of four glycoproteins, called ZP1–4 (Lefievre et al., 2004). During folliculogenesis, ZP2 and ZP3, acting as building blocks, polymerize and incorporate into fibrils that form an extracellular matrix around the growing oocytes, while ZP1 acts as a cross-linker of the individual fibrils (Wassarman, 2008). ZPD is a common element of ZP proteins that consists of two separated domains marked as ZP-N and ZP-C. It is responsible for mediating homodimerization of intracellular ZP3 (dormant status) as well as the heteropolymerization of extracellular ZP2 and ZP3 and their incorporation into ZP fibrils (Bokhove and Jovine, 2018). Thr189_Gly193 is located within the ZP-C subdomain, and its deletion might affect formation and stabilization of dimeric ZP3, thereby leading to the degradation of ZP3 proteins within cells.

The zona matrix acts as a structural basis supporting the formation of gap junctions, a key structure for signal transduction between the growing oocyte and the surrounding cumulus cells (Wassarman, 2008). Our recent study presented the morphological evidence showing an abnormal ZP assembly and antral-follicular development in EFS patients with biallelic *ZP1* mutations (Dai et al., 2019). Here, similar anomalies in ZP assembly were observed in the EFS patient carrying a heterozygous *ZP3* mutation, but degenerated oocytes were observed in the earlier stages and pre-antral follicles. However, dispersive ZP3 signals were detected surrounding the patient's oocyte within a secondary follicle (Figure 2N), which might result from secretion and accumulation of the relict ZP3 protein during follicle growth. We speculated that the shortage of secreted ZP3 protein and the defective ZPD in mutant ZP3 affect the ZP assembly, which further leads to a defective gap junction and cumulus–oocyte complex organization, eventually resulting in oocyte degeneration and empty follicle-like structures.

Interestingly, our earlier study showed that patients carrying homozygous *ZP2*-truncating mutations produced mature oocytes with a thin ZP that lacks ZP2, and this ZP2-null matrix sustained until the blastocyst stage (Dai et al., 2019b). ZP4 is a pseudogene in mice, while it is expressed in humans and may substitute ZP2 in forming a zona matrix as reported in mice (Avella et al., 2014). Collectively, these observations suggest a key role of ZP3 in ZP assembly and support the opinion that the absence of ZP3 cannot be overcome (Fahrenkamp et al., 2020).

In conclusion, we reported a novel heterozygous *ZP3* deletion associated with EFS and female infertility. We also showed the abnormal ZP assembly and pre-antral follicular development. Our findings expand the mutational spectrum associated with human EFS, and provide new insights into its pathogenesis.

## Data Availability Statement

The raw data supporting the conclusions of this article will be made available by the authors, without undue reservation, to any qualified researcher.

## Ethics Statement

The studies involving human participants were reviewed and approved by Ethics Committee of the Reproductive and Genetic Hospital of CITIC-Xiangya. The patients/participants provided their written informed consent to participate in this study.

## Author Contributions

CD, GLu, and GLin conceived and designed the study. YC, ZW, WH, and YW carried out the experiments. JD, SC, and FG provided the clinical samples. YC wrote the manuscript. CD and GLin critically commented on and edited the manuscript. All authors read and approved the final version of the manuscript.

## Conflict of Interest

The authors declare that the research was conducted in the absence of any commercial or financial relationships that could be construed as a potential conflict of interest.
